# Editor’s page

**DOI:** 10.21542/gcsp.2025.34

**Published:** 2025-06-30

**Authors:** Magdi H Yacoub

**Affiliations:** Aswan Heart Centre, Aswan, Egypt; Imperial College, London, UK

This month marks 13 years of the journal’s existence. In this period the journal’s policy has been devoted to present the latest developments in cardiology and cardiac surgery in an easily accessible form, with an emphasis on global issues and equity in healthcare delivery. Consistent with this policy, and starting from the next issue, we are delighted to add a new section to the journal entitled “Surgical Techniques” to present in-depth, well-illustrated operations with new modifications, their rationale, and their outcomes. We particularly welcome submissions that combine text, images and video.

We also welcome two new associate editors to the journal, Amr Alsalakawy and Hatem Hosny


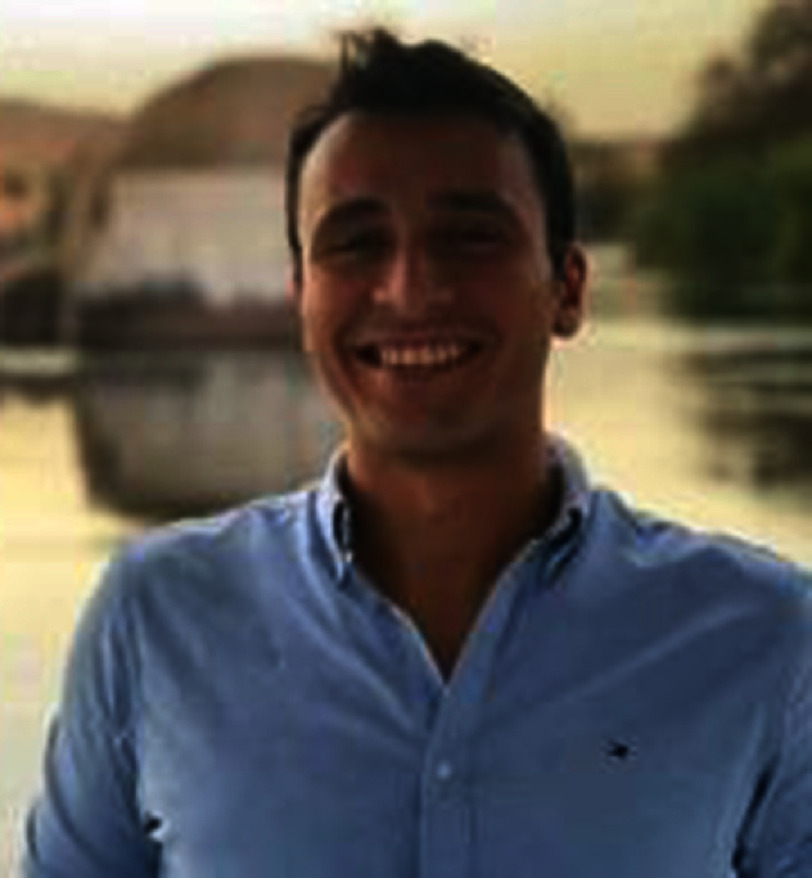


**Amr Alsalakawy**

Dr. Amr Alsalakawy is a cardiac surgeon at the Aswan Heart Centre, with experience in both congenital and adult complex cardiac surgery. He is passionate about surgical innovation that not only advances clinical practice but also makes a meaningful difference in patients’ lives, particularly in underserved communities.


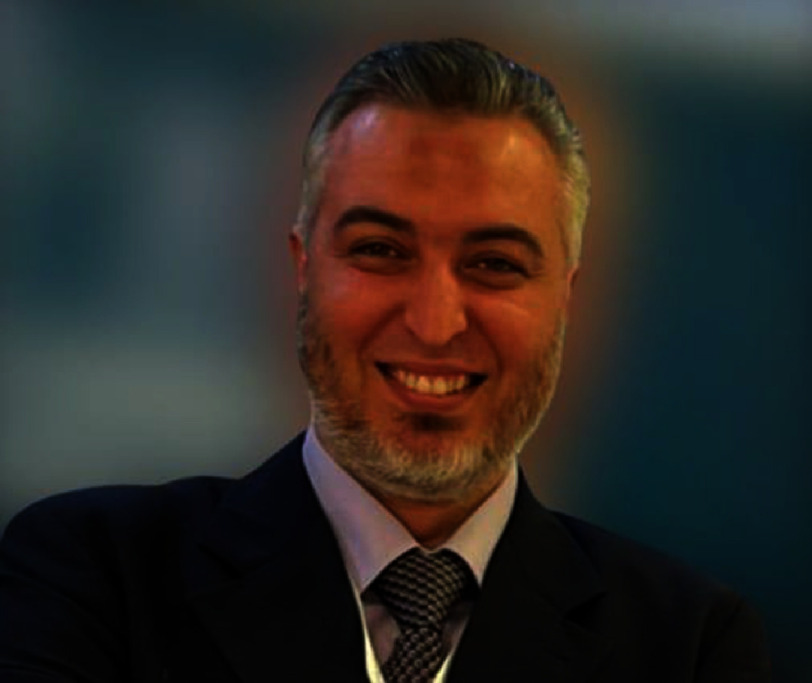


**Hatem Hosny**

Hatem Hosny, FRCS, FEBCTS (Cardiac), FEBCTS (Congenital) is a consultant cardiac surgeon at the Aswan Heart Centre with special interests in congenital heart disease and medical education.

